# The Antioxidant and Safety Properties of Spent Coffee Ground Extracts Impacted by the Combined Hot Pressurized Liquid Extraction–Resin Purification Process

**DOI:** 10.3390/molecules23010021

**Published:** 2017-12-22

**Authors:** María Salomé Mariotti-Celis, Maximiliano Martínez-Cifuentes, Nils Huamán-Castilla, Mario Vargas-González, Franco Pedreschi, José Ricardo Pérez-Correa

**Affiliations:** 1 Programa Institucional de Fomento a la Investigación, Desarrollo e Innovación, Universidad Tecnológica Metropolitana, Ignacio Valdivieso 2409, P.O. Box 9845, Santiago 8940577, Chile; mmartinez@utem.cl; 2 Chemical and Bioprocess Engineering Department, School of Engineering, Pontificia Universidad Católica de Chile, Vicuña Mackenna 4860, P.O. Box 306, Santiago 7820436, Chile; nlhuaman@uc.cl (N.H.-C.); fpedreschi@ing.puc.cl (F.P.); perez@ing.puc.cl (J.R.P.-C.); 3 Escuela de Ingeniería Agroindustrial, Universidad Nacional de Moquegua, Prolongación calle Ancash s/n, Moquegua 18001, Peru; 4 Departamento de Química, Universidad Tecnológica Metropolitana, Las Palmeras 3360, P.O. Box 9845, Santiago 7800003, Chile; mvargas360@hotmail.com

**Keywords:** polyphenols, hydroxymethylfurfural, feruloylquinic acid, epicatechin

## Abstract

Hot pressurized liquid extraction has been used to obtain polyphenols; however, its operating conditions can generate hydroxymethylfurfural, a potential human carcinogen. The addition of ethanol can reduce process temperatures and retain extraction efficiencies, but the ethanol may reduce the recovery of polyphenols in the subsequent purification stage, affecting the antioxidant properties of the extracts. This study evaluates a combined hot pressurized liquid extraction—resin purification process to obtain polyphenol extracts from spent ground coffee reduced in hydroxymethylfurfural. A multifactorial design was developed to determine the combined effect of the extraction (ethanol content: 0–16% and temperature: 60–90 °C) and purification (ethanol: 60–80%) conditions on some chemical properties of the extracts. The highest recovery of polyphenols (~8 mg GAE/g dry coffee solids) and reduction of hydroxymethylfurfural (95%) were obtained at 90 °C and 16% of ethanol during extraction and 80% of ethanol during purification. These operating conditions retained the antioxidant capacity of the crude extract between 60% and 88% depending on the determination method and recovered 90, 98, and 100% of 4-feruloylquinic acid, epicatechin, and 5-feruloylquinic acid, respectively after purification. The combined process allows differential polyphenols’ recovery and enhances the safety of the extracts. Our computational chemistry results ruled out that the overall selectivity of the integrated process was correlated with the size of the polyphenols.

## 1. Introduction

Coffee is one of the major contributors to the dietary intake of polyphenols worldwide [1,2]; however, its high worldwide consumption (~9.6 million TM/year) has generated large amounts of residues which represent serious environmental problems [3]. The solid residue obtained after processing roasted coffee with hot water is known as spent coffee grounds (SCG), which contain important quantities of polyphenols, including the major caffeic and chlorogenic acids as well as epicatechin [4,5,6]. These compounds have shown several biological activities, including antiproliferative, antioxidant, and antimicrobial effects [7]. Additionally, their intake has been inversely associated with the risk of chronic diseases such as cardiovascular disorders and cancer [7].

Conventional extraction has been used for obtaining polyphenols from agroindustrial byproducts; however, the organic solvents commonly used in this process (e.g., methanol and acetone, among others) are questionable due to their high toxicity and damage to the environment [8].

Hot pressurized water extraction (HPWE) is a green alternative technology to obtain polyphenols from vegetal matrices, which is characterized by its short extraction times (5–20 min), subcritical temperatures (110–300 °C) and high pressures (10–15 MPa) [9]. Under these conditions, the physical and chemical properties of water change dramatically, improving the efficiency and selectivity of the extraction [10]. The increasing temperature facilitates the analyte diffusion and favors the mass-transfer kinetics phenomenon. Additionally, the high temperature produces the weakening of hydrogen bonds of water, decreasing its dielectric constant (“measure of polarity”). Thus, water can be used as an alternative to dissolve medium-polar and low-polar organic compounds as it is a significant fraction of the polyphenols [11]. Similarly, in hot pressurized liquid extraction (HPLE) the use of ethanol as co-solvent (temperature: 160–190 °C; co-solvent: 25–75%) improves the recovery of total polyphenol content (TPC: up to 88 mg GAE/g) [12].

Nevertheless, the high temperatures applied at subcritical operating conditions would favor the formation and extraction of some potential human carcinogens associated with the Maillard reaction, such as acrylamide and hydroxymethylfurfural (HMF) [13,14]. HMF is considered to be a good indicator of the presence of Maillard toxic compounds in thermally treated foods, not only because its content directly relates with the occurrence of acrylamide and other furans [15,16,17,18] but also since its quantification in food matrixes is significantly easier and cheaper than the other Maillard compounds [13,14,19]. Therefore, its determination, as one of the responses to optimize the polyphenol extraction, can help enhance the safety of the process [10].

In previous research, we found that the addition of ethanol as co-solvent during hot pressurized liquid extraction (HPLE) enables a decrease in the extraction temperature (from 130 °C to 90 °C) preserving 90% the total polyphenols content of the extracts [10]. This change in the HPLE operating conditions mitigated the HMF formation during the extraction stage. However, for raw materials such as SCG that contain considerable amounts of HMF before the extraction, other options should be assessed [20,21].

In this sense, a subsequent purification process which promotes the selective separation of polyphenols, could enhance the safety of the extracts [22]. However, the presence of ethanol in the extraction stage may reduce the recovery of polyphenols at the subsequent resin purification step (RP), since the elution of these compounds is carried out with an ethanol/water mixture.

Adsorption/desorption in columns packed with synthetic macroporous resins is frequently used to purify polyphenol extracts, due to its simple operation, high efficiency, relatively low cost, low environmental impact, and easy regeneration [23,24]. It consists of three steps: adsorption of polyphenols, washing of the resin with water to eliminate undesirable compounds, and desorption of polyphenols, normally with an ethanol/water mixture as the eluent.

HP-20 is a polyaromatic adsorbent resin (polystyrene-divinylbenzene) without polar groups, suitable for recovering hydrophobic compounds; it is widely applied for processing foods and beverages [13,14]. The adsorption/desorption of polyphenols (adsorbates) depends on the hydrophobic interactions (e.g., van der Waals forces) between them and the functional groups of the macroporous resins [25]. All these forces can contribute to the intensity of the interaction between the polyphenols and the resin although these interactions can be altered by the properties of the solvent after a given polarity threshold [26]. The adsorption/desorption phenomenon is also generally affected by the specific surface and porosity of the resin as well as by the particle size of the specific compound [25]. Adsorption could occur on the surface or inside the particle. Intra-particular adsorption is usually irreversible, decreasing the polyphenols’ recovery [27].

A better understanding of the physical and chemical interactions that govern the extraction and purification of polyphenols can contribute to optimize this integrated process. In this sense, the use of computational chemistry tools is an attractive option to determine some features of polyphenols that could help elucidate the main causes governing the differential separation [28,29,30,31].

This research assesses the effect of the operating conditions of a combined hot pressurized liquid extraction–resin purification process (HPLE–RP) on the differential recovery of polyphenols and HMF from SCG.

## 2. Materials and Methods

Following a full factorial experimental design, SCGs were extracted by HPLE with the addition of small amounts of ethanol at different extraction temperatures. The extracts were subsequently purified (RP) using ethanol/water solutions as a desorption eluent. Their total polyphenol content (TPC) and HMF concentration were quantified to select the best operating conditions (within the range assessed in this study), defined as those in which the highest recovery of polyphenol compounds and the lowest HMF content is reached. Then, the antioxidant capacity (AOC) of the crude and purified extracts obtained at the best operating conditions was determined using the 2,2-diphenyl-1-picrylhydrazyl (DPPH) and the oxygen radical absorbance capacity (ORAC) methods and compared with those obtained using a HPLE–RP process. Additionally, the polyphenols’ profile of the best purified extract was determined and analyzed using computational chemistry.

### 2.1. Chemicals and Analytic Reagents

HPLC grade reagents, standards, and solvents (Sigma Aldrich, Steinheim, Germany) were used in the extraction and chemical analyses. These include Folin–Ciocalteu reagent, Carrez solution I, Carrez solution II, dimethylaminocinnamaldehyde (DMAC; F.W. 175.23), sodium carbonate, sodium chloride, sodium hydroxide, ammonium hydroxide, standards (protocatechualdehyde, p-hydroxybenzaldehyde, catechin, epicatechin, HMF, as well as gallic, p-coumaric, gentisic, ferulic, caffeic, syringic, vanillic, and protocatechuic acids), and solvents (acetone, methanol, acetonitrile, formic acid, hydrochloric acid, acetic acid, methanol, and ethanol).

### 2.2. Spent Coffee Grounds (SCG)

SCGs (*Coffea arabica* L.) were obtained, after preparing the coffee with water at 80 °C and 4 min of repose, using water in a Inox Oster filter coffeemaker (model OEMP50, Sunbeam Products, Inc., Boca Raton, FL, USA). Before extraction experiments, SCGs were dried at 40 °C during 15 h approx., until reaching a moisture content of 36.8% ± 0.1% (*w*/*w*). Then, dried SCG samples were reduced to a particle size lower than 1 mm diameter by an Oster blender (Sunbeam Products, Inc., Boca Raton, FL, USA) and immediately frozen (−20 °C) until extraction (less than two months).

### 2.3. Hot Pressurized Liquid Extraction

SCG (~5 g) homogenized samples were subjected to HPLE in an Accelerated Solvent Extractor (ASE 150, Dionex, Sunnyvale, CA, USA) according to Vergara-Salinas et al. [32]. Polyphenol extraction was carried out at different extraction temperatures and ethanol contents, following a multifactorial experimental design described below. An additional SCG extraction was carried out using pure water (HPWE) at 200 °C. After extraction, crude extracts were collected and stored in amber vials at −20 °C prior to the subsequent RP process and chemical analysis.

### 2.4. Macroporous Resin Purification of SCG Extracts

SCG extracts were purified using a polystyrene column packed (Ø: 25 mm; h: 100 mm) with ~10 g of HP-20 resin (Diaion, Tokyo, Japan). This resin was selected in preliminary tests performed at our laboratory, due to its high SCG polyphenols adsorption capacity compared to other resins (Sepabeads SP850, Amberlite XAD7). For polyphenols adsorption, 100 mL of SCG extract were passed through the resin with a flow rate of 5 mL/min. Then, polyphenol desorption was carried out using different ethanol/water solutions as eluents at a rate of 5 mL/min. The adsorption/desorption polyphenol experiments were performed at 30 °C. Finally, purified extracts were stored at −20 °C until chemical analysis.

### 2.5. Determination of the Total Polyphenol Content (TPC)

The TPC of SCG extracts was determined by a Folin-Ciocalteu assay [33] (Spectrometer UV 1240 Shimadzu, Kioto, Japan). Results were expressed as g of gallic acid equivalent (GAE) per g of dry spent coffee.

### 2.6. Determination of Antioxidant Capacity

The antioxidant capacity of SCG extracts was determined by DPPH and an improved version of the ORAC assay developed by Ou et al. [34]. 

DPPH was performed by spectrophotometry (Spectrometer UV 1240, Shimadzu, Kioto, Japan) according to the free radical 2,2-diphenyl-1-picrylhydrazyl method (DPPH) [35]. The efficient concentration of extracts, which is the concentration necessary to inhibit 50% the absorption of DPPH (EC50; mg/mL), was determined in triplicate and expressed as mg of Trolox equivalents per g of dry SCG (mg TE/g). 

The ORAC assay was carried out in a PerkinElmer 2030 Multilabel Reader with 96-well black plates [34]. The Trolox equivalent molar concentrations of the samples were calculated using a linear regression equation between the Trolox concentration and the corresponding net AUC [34]. To compare the antioxidant activity of the extracts, we decided to calculate the relative ORAC values as mg of Trolox equivalents present in 1 g of dry SCG.

### 2.7. Quantification of HMF

HMF concentrations of the extracts were measured by HPLC-DAD (Thermo Scientific Dionex Ultimate 3000, Waltham , MA, USA) equipped with a reverse phase AcclaimTM 120 C18 column (5 µm 120 Å 4.6 × 150 mm) according to the methodology of Toker et al. [36]. Analyses were performed in triplicate and results were expressed in mg of HMF per g of dry spent coffee grounds.

### 2.8. Determination of the Polyphenol Profile

The polyphenol profile of the extracts obtained at optimal conditions was determined by solid phase extraction on polymeric cartridges and liquid chromatography with diode array detection (SPE/HPLC-DAD) according to the methodology of Del Alamo et al. [37].

### 2.9. Experimental Design and Statistical Analyses

A full factorial experimental design (3 × 4 × 3) with three replicates was developed in order to investigate the effect of extraction temperature (at three levels: 60, 75, and 90 °C), co-solvent content (at four levels: 0%, 5.3%, 10.5%, and 16.0% of ethanol) and eluent concentration (at three levels: 60%, 70%, and 80% of ethanol) over the total polyphenol and HMF content of the SCG extracts obtained using an integrated HPLE–RP process. The experimental design consisted of 36 combinations of the independent variables (extraction temperature, co-solvent content and eluent ethanol concentration) performed in random order.

The HPLE–RP process and chemical analysis were performed in triplicate with the data presented as mean and coefficient of variation (CV). To study the effects of the studied factors and their interactions on purification performance, analysis of variance (ANOVA), and least significant difference tests were applied to the response variables with a significance of *p* ≤ 0.05. The program Statgraphics Plus for Windows 4.0 (Statpoint Technologies, Inc., Warrenton, VA, USA) was used for statistical analysis.

### 2.10. Computational Chemistry

Calculations were carried out using the Gaussian 09 [38] program package (Gaussian, Inc., Wallingford, CT, USA) running in a Microsystem (Sun Microsystem, Menlo Park, CA, USA). Geometries were calculated at density functional theory (DFT) M062x/6-31+G(d,p) level. No imaginary vibrational frequencies were found at the optimized geometries, which indicate that they are true minima of the potential energy surface.

## 3. Results and Discussion

### 3.1. Impact of the Operating Conditions of a HPLE-RP Combined Process on the Chemical Composition and Antioxidant Properties of SCG Extracts

The impact of the operating conditions on the TPC and HMF contents of the purified extracts obtained by a HPLE–RP combined process are shown in Table 1. The best operating conditions found were 90 °C and 16% of ethanol during extraction and 80% of ethanol during purification in which the TPC and HMF contents of SCG extracts were 8.46 mg/g dry coffee solids and 1.82 µg/g dry coffee solids, respectively (Table 1).

**Figure 1:**
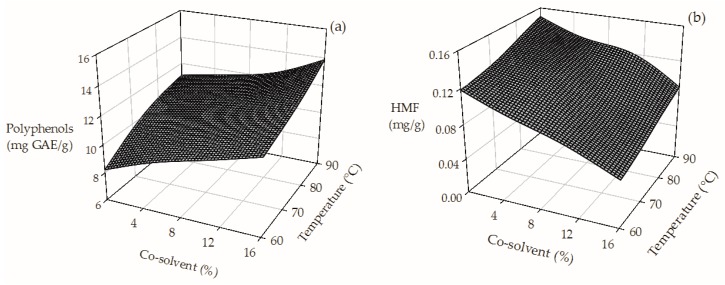
Effect of the operating conditions on the TPC (**a**) and HMF (**b**) content of SGC extracts obtained by HPLE.

An increase in the operating conditions during HPLE (temperature: from 60 °C to 90 °C and co-solvent concentration from 0% to 16%) improved almost 70% the extraction of total polyphenols (from 8.21 to 13.87 mg GAE/g dry SCG) as can be observed in Figure 1a. A higher extraction temperature favors the mass transfer of polyphenols from the raw material to the extraction solvent. Additionally, because of their polarity, the higher the co-solvent content, the higher the total polyphenol content of the extracts [22]. In our study, we found that the SCG crude extracts contain considerable amounts of HMF (Table 1). Roasting of coffee beans is commonly performed at extremely high temperatures (~220 °C), triggering the generation of Maillard compounds such as HMF [20,21]. However, we observed that the addition of ethanol, up to 16% during HPLE, decreased ~50% the HMF content of the SCG extracts (Figure 1b). The addition of a non-polar co-solvent in the HPLE process reduced the polarity of the medium affecting the solubility of HMF and disfavoring its extraction [39].

**Figure 2:**
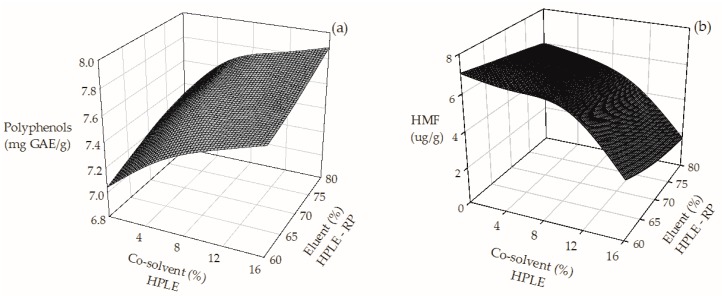
Effect of co-solvent addition on the TPC (**a**) and HMF (**b**) content of SGC extracts obtained by a combined HPLE–RP process.

Successful formulations of nutraceutical and functional ingredients using SCG extracts will depend not only on their antioxidant capacity, but also on their purity as well as their safety. Consequently, the application of a subsequent RP stage was assessed. Increasing the ethanol content of the eluent (from 60% to 80%) during RP improved the recovery of polyphenols (Figure 2a) by 20%. On the contrary, higher ethanol contents reduced HMF in the purified extract up to 95% (Figure 2b). These results indicate that, at the best operating conditions found, the combined HPLE–RP process improves the selective recovery of polyphenols, since HMF is practically eliminated.

**Figure 3:**
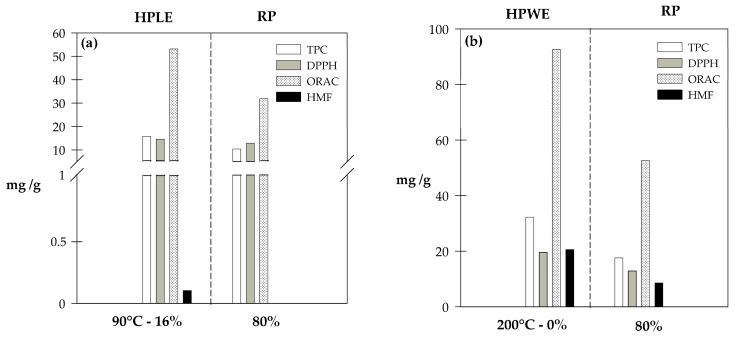
Antioxidant potential of SCG extracts obtained at the best HPLE-RP conditions (**a**) and at HPWE-RP conditions (**b**). DPPH and ORAC are expressed as mg Trolox equivalent (ET) per g of dry SCG.

On the other hand, the integration of the RP stage after the extraction process decreased the TPC and the AOC, both in the HPLE (TPC: 34%, DPPH: 12% and ORAC: 40%) and the HPWE processes (TPC: 46%, DPPH: 34% and ORAC: 43%) as seen in Figure 3a,b, respectively. However, different AOC responses were observed for DPPH and ORAC (Figure 3a,b). It can be attributed to some intrinsic characteristics of the assays as well as the variations in the polyphenol profile of crude and purified extracts. ORAC analysis evaluates the AOC against small radicals, while DPPH is a bulky radical which generates steric inaccessibility, specifically for polyphenols that possess strong antioxidant activities. This size difference increases the probability of reaction when the ORAC method is applied. On the contrary, when the AOC of extracts is determined by DPPH, the system may react slowly or may even be inert to DPPH [40]. Additionally, some polyphenols present different values of ORAC and DPPH. Therefore, if the polyphenol profile of the extracts changes, their ORAC and DPPH values do not necessarily change in the same magnitude [41,42].

Although HPWE–RP could obtain an extract with higher AOC and TPC than the best HPLE–RP operating conditions found in this study, the HPWE–RP extract contained considerable amounts of HMF (~7.6 mg/g dry SCG), due to the high extraction temperature (200 °C) applied [43].

### 3.2. Chemical Composition Changes Experimented by the SCG Extracts during the Best HPLE-RP Operating Conditions

The polyphenols profile of the extract obtained at the best HPLE–RP operating conditions significantly changed after the RP process. Under these conditions, a differential recovery was observed. Some polyphenols presented recoveries over 90% in the purified extract (4-feruloylquinic acid, 5-feruloylquinic acid, and epicatechin), others were not recovered at all (gallic acid, caffeic acid, 5-p-coumaroylquinic acid, 3,5-dicaffeoylquinic acid, and 3-feruloyl-4-caffeoylquinic acid) and some were partially recovered (3-caffeoylquinic acid, 5-caffeoylquinic acid, 3-feruloylquinic acid, 3-feruloyl-4-caffeoylquinic acid, and 3,4-dicaffeoylquinic acid), as seen in Table 2.

The interactions between the polyphenols and the resin, as well as the effect of the polarity of the solvent over them, can contribute to explain the differential recovery obtained. HP-20 is a polyaromatic adsorbent resin without polar groups. It is reasonable to assume that, in addition to van der Waals forces, π-stacking can occur between the aromatic rings of the polyphenols and the aromatic rings in the surface of the resin [44,45]. However, all these forces can be altered by the properties of the solvent after a given polarity threshold [46,47].

To assess a possible association between molecular size and differential recovery of polyphenols, the molecular dimensions of all compounds identified in the profile were determined by quantum chemical calculation (DFT M062x/6-31+G(d,p) level). From optimized geometries for each compound, at the level indicated above, the largest diameters and molecular volumes were determined. We found no correlation between these molecular dimensions and the observed polyphenol differential recovery (Table 2). Additionally, Table 2 shows no correlation between the number of aromatic rings in the molecules and their overall recovery in the integrated process.

The analyzed SCG polyphenols present specific interactions with the resin or fulfil specific topological requirements that explain the high selectivity of the optimized process. We hypothesized that, even under the best operating conditions, the adsorption polarity threshold of some SCG polyphenols was not reached. Therefore, even though the co-solvent addition level was increased, the hydrophobic interactions were not affected, and the polarity of the HPLE extracts was still appropriate to efficiently recover these compounds. It seems, however, that for the SCG polyphenols not found in the purified extract, either the polarity of the optimum extraction solvent altered their interaction with the resin, or they were irreversibly adsorbed in the pores of the resin. To elucidate the true mechanism, further experiments are necessary, which are beyond the scope of this study.

## 4. Conclusions

The use of ethanol as co-solvent during HPLE improves the extraction efficiency of polyphenols at moderate temperatures and disfavors the recovery of the HMF from SCG. Ethanol addition up to 16% in the extraction stage shows overall recoveries over 90% for 4-feruloylquinic acid, 5-feruloylquinic acid, and epicatechin, although some polyphenols, such as gallic acid, caffeic acid, 5-*p*-coumaroylquinic acid, 3,5-dicaffeoylquinic acid, and 3-feruloyl-4-caffeoylquinic acid, were not recovered in the purified extract. It was ruled out that the high polyphenols selectivity observed in the RP process was related to the size of the molecules. Additionally, the best HPLE-RP operating conditions eliminated 95% of the HMF from the purified extract (1.82 µg HMF/g dry SCG) retaining the antioxidant capacity of the crude extract between 60% and 88%, depending on the determination method (DPPH and ORAC, respectively). Extractions with pure water at high temperatures (200 °C) produce purified extracts with undesirable high contents of HMF (8.69 mg HMF/g dry SCG).

## Figures and Tables

**Table 1:** Chemical characterization of SCG extracts obtained by a combined HPLE–RP process.

Temperature (°C)-Ethanol (%)	TPC (mg GAE/g)		HMF (mg/g)		Eluent%	TPC (mg GAE/g)		HMF (µg/g)	
	Mean	CV	Mean	CV		Mean	CV	Mean	CV
60–0	8.21 ^a^	0.01	0.12 ^a^	0.09	60	6.78 ^a^	0.01	6.23 ^l^	0.01
					70	6.98 ^b^	0.02	5.46 ^h,i^	0.01
					80	7.20 ^e,f^	0.03	5.17 ^h^	0.02
60–5.3	9.51 ^b^	0.02	0.10 ^b^	0.07	60	6.81 ^a^	0.03	5.91 ^k^	0.01
					70	7.07 ^o^	0.02	5.25 ^h^	0.02
					80	7.19 ^d,e,f^	0.01	4.81 ^g^	0.01
60–10.6	10.44 ^c^	0.00	0.08 ^c^	0.09	60	7.14 ^c,d,e^	0.04	5.51 ^i^	0.00
					70	7.22 ^f,g^	0.01	3.97 ^f^	0.01
					80	7.34 ^i,j^	0.01	3.21 ^d^	0.02
60–16	11.43 ^d^	0.01	0.05 ^d^	0.06	60	7.29 ^g,h,i^	0.01	3.12 ^d^	0.02
					70	7.45 ^k,l,m^	0.02	2.11 ^b^	0.01
					80	7.52 ^n,o^	0.02	1.57 ^a^	0.03
75–0	10.18 ^c^	0.02	0.13 ^a^	0.09	60	7.11 ^c,d^	0.01	6.88 ^n^	0.00
					70	7.25 ^f,g,h^	0.02	6.81 ^n^	0.00
					80	7.33 ^h,i,j^	0.01	5.92 ^k^	0.00
75–5.3	10.81 ^c^	0.01	0.12 ^a^	0.07	60	7.45 ^k,l,m^	0.01	6.53 ^m^	0.00
					70	7.51 ^m,n,o^	0.02	6.35 ^l,m^	0.00
					80	7.57 ^o,p^	0.01	5.53 ^i,j^	0.00
75–10.6	11.55 ^d^	0.01	0.09 ^b,c^	0.02	60	7.47 ^l,m,n^	0.02	5.69 ^j^	0.01
					70	7.54 ^n,o,p^	0.01	4.40 ^f^	0.02
					80	7.60 ^p,q^	0.01	3.92 ^e,f^	0.01
75–16	12.42 ^e^	0.01	0.07 ^d^	0.03	60	7.55 ^o,p^	0.01	3.28 ^e^	0.02
					70	7.61 ^p,q^	0.01	2.26 ^c^	0.02
					80	7.67 ^q,r^	0.01	1.70 ^a^	0.01
90–0	11.07 ^c^	0.00	0.15 ^e^	0.09	60	7.24 ^f,g^	0.02	8.04 ^q^	0.00
					70	7.39 ^j,k^	0.02	7.33 ^o,p^	0.00
					80	7.42 ^k,l^	0.01	6.89 ^n^	0.00
90–5.3	11.66 ^d^	0.02	0.13 ^a^	0.05	60	7.73 ^r^	0.01	7.46 ^o,p^	0.00
					70	8.10 ^t,u^	0.02	6.90 ^n^	0.00
					80	8.24 ^v^	0.01	6.13 ^l^	0.00
90–10.6	12.35 ^e^	0.01	0.12 ^a,b^	0.08	60	7.91 ^s^	0.02	7.16 ^o^	0.01
					70	8.11 ^t,u^	0.01	6.54 ^m^	0.02
					80	8.41 ^w^	0.02	5.92 ^k^	0.01
90–16	13.87 ^f^	0.01	0.09 ^b,c^	0.01	60	8.05 ^t^	0.02	3.43 ^e^	0.01
					70	8.16 ^u,v^	0.02	2.39 ^c^	0.01
					80	8.46 ^w^	0.03	1.82 ^b^	0.01

Polyphenol and HMF contents are expressed as mg per g of dry SCG; CV: Coefficient of variation. Different letters between files, show significant differences (*p* ≤ 0.05) between the extraction treatments applied.

**Table 2:** Physic and chemical properties of SCG extracts obtained under the best operating conditions of a combined HPLE–RP process.

Molecule	Mw (g/mol)	Largest Diameter (Å)	Volume (Å^3^)	Aromatic Rings	HPLE µg/g	HPLE–RP µg/g
5-Feruloylquinic Acid	368.34	14.16	474.26	1	2.65	2.65
(±) Epicatechin	290.27	10.36	381.92	2	429.38	422.14
4-Feruloylquinic Acid	368.34	13.58	447.54	1	7.57	6.83
3,4-DiCaffeoylquinic Acid	516.45	15.24	508.33	2	4.58	1.68
3-Feruloylquinic Acid	368.34	12.85	444.35	1	25.39	1.13
3-Caffeoylquinic Acid	354.31	12.84	410.75	1	16	0.72
3-Feruloyl-4-Caffeoylquinic Acid	530.48	15.24	548.21	2	1.7	0.64
5-Caffeoylquinic Acid	354.31	14.09	374.67	1	29.99	0.44
5-p-Coumaroylquinic Acid	338.31	14.22	383.34	1	3.91	ND
Gallic acid	170.12	6.87	199.50	1	1.11	ND
3,5-DiCaffeoylquinic Acid	516.45	19.42	627.97	2	2.19	ND
4,5-DiCaffeoylquinic Acid	516.45	14.88	563.67	2	0.71	ND
